# Direct biomolecule discrimination in mixed samples using nanogap-based single-molecule electrical measurement

**DOI:** 10.1038/s41598-023-35724-1

**Published:** 2023-06-05

**Authors:** Jiho Ryu, Yuki Komoto, Takahito Ohshiro, Masateru Taniguchi

**Affiliations:** 1 SANKEN, Osaka University, 8-1 Mihogaoka, Ibaraki, Osaka 567-0047 Japan; 2 Artificial Intelligence Research Center, Osaka University, Ibaraki, Osaka 567-0047 Japan; 3 Integrated Frontier Research for Medical Science Division, Institute for Open and Transdisciplinary Research Initiative (OTRI), Osaka University, Ibaraki, Osaka 567-0047 Japan

**Keywords:** Molecular electronics, Nanosensors

## Abstract

In single-molecule measurements, metal nanogap electrodes directly measure the current of a single molecule. This technique has been actively investigated as a new detection method for a variety of samples. Machine learning has been applied to analyze signals derived from single molecules to improve the identification accuracy. However, conventional identification methods have drawbacks, such as the requirement of data to be measured for each target molecule and the electronic structure variation of the nanogap electrode. In this study, we report a technique for identifying molecules based on single-molecule measurement data measured only in mixed sample solutions. Compared with conventional methods that require training classifiers on measurement data from individual samples, our proposed method successfully predicts the mixing ratio from the measurement data in mixed solutions. This demonstrates the possibility of identifying single molecules using only data from mixed solutions, without prior training. This method is anticipated to be particularly useful for the analysis of biological samples in which chemical separation methods are not applicable, thereby increasing the potential for single-molecule measurements to be widely adopted as an analytical technique.

## Introduction

**Figure 1:**
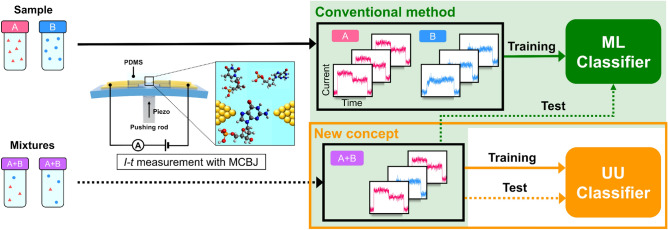
Flow chart of single-molecule classification. For single-molecule current measurements, the sample solutions were injected into a PDMS well, and the chips were bent with a finely controlled push bar with a piezoelectric device to form a nanogap, after which the current was measured. The green box represents the conventional method, while the orange box represents the new concepts. The solid lines show the process for each individual sample, and the dashed lines show the process for the mixture.

The direct measurement of complex samples offers advantages such as time and cost savings by minimizing the sample preparation steps and sample loss, while also enabling the detection of a wide range of molecules. Single-molecule measurement is attracting attention as a novel molecular detection/quantification measurement method because in this method, a molecule between nanoelectrodes is directly measured^1–3^. In the break junction method^4–7^, a single-molecule electrical measurement method, a metal nanogap is formed by repeatedly breaking and forming junctions. A single molecule is detected by measuring the tunneling current that occurs when a molecule passes through the nanogap. Single-molecule measurements are being actively researched for the development of molecular devices^2,8–13^. Since Di Ventra’s group theoretically proposed the potential for DNA and RNA sequencing, single-molecule measurements have received significant attention as an analytical method owing to their high throughput, low detection limit, and the ability to conduct measurements with no preprocessing steps^3,14,15^. To date, our group has reported conductance measurements of DNA and RNA nucleobases and demonstrated the applicability of single-molecule measurements as an analytical method^16–18^. The target molecules are not limited to DNA and RNA, and can be extended to various molecules such as amino acids^19,20^, peptides^21,22^, proteins^23–25^, neurotransmitters^26^, glucose^27^, and NADH^28^. Furthermore, the measurement targets are not limited to biomolecules. Single-molecule measurements are expected to have a wide range of applications; for example, the potential of detecting explosives^29^. Although the conductance of different molecules can be measured with single-molecule measurements, single-molecule conductance is highly variable^30–33^. Therefore, the statistical evaluation of single-molecule signals is essential for reliable molecular identification. Most typical conductance histograms-based analysis provides only statistical conductance information on single-molecule conductance. The overlapping of conductance histograms results in a low accuracy in single-molecule discrimination. The application of machine learning to single-molecule measurements is a promising method to address these issues. Machine learning-based analysis has improved the discrimination accuracy of single-molecule measurements^26,34–38^. However, conventional machine-learning approaches require training data obtained from solutions containing only one chemical species for every target molecule. Considering the application of single-molecule measurements for detecting biomolecules or specific targets, preparing a reference containing only one sample from a solution containing impurities for all molecules is occasionally difficult. However, preparing samples with varying concentrations of the target molecules in impure solutions can be comparatively easier. For example, by promoting or inhibiting the emission of the target in biological samples or adding a reference molecule in a sample solution. Even if a solution containing only a specific target molecule can be measured, the machine-learning classifier built with the training data may not be applicable to the samples because the measurement environment of the training data may be different from that of the sample. From these reasons, the development of a method for direct discrimination from mixed samples without single-species target samples, represents a significant advancement in the field of single-molecule measurements. The approach has significant potential in providing insights into the detection of biological molecules and other targets in complex samples. Herein, the aim of this study was the development of an analytical method for identifying molecules based only with mixed solutions. As shown in Fig 1, targeting dGMP and dTMP, which are already known to be identifiable by pure solution single-molecule measurements and conventional machine learning-based analysis, we developed a method to determine the concentration ratio of mixed solutions from their mixtures only.

## Results and discussion

**Figure 2:**
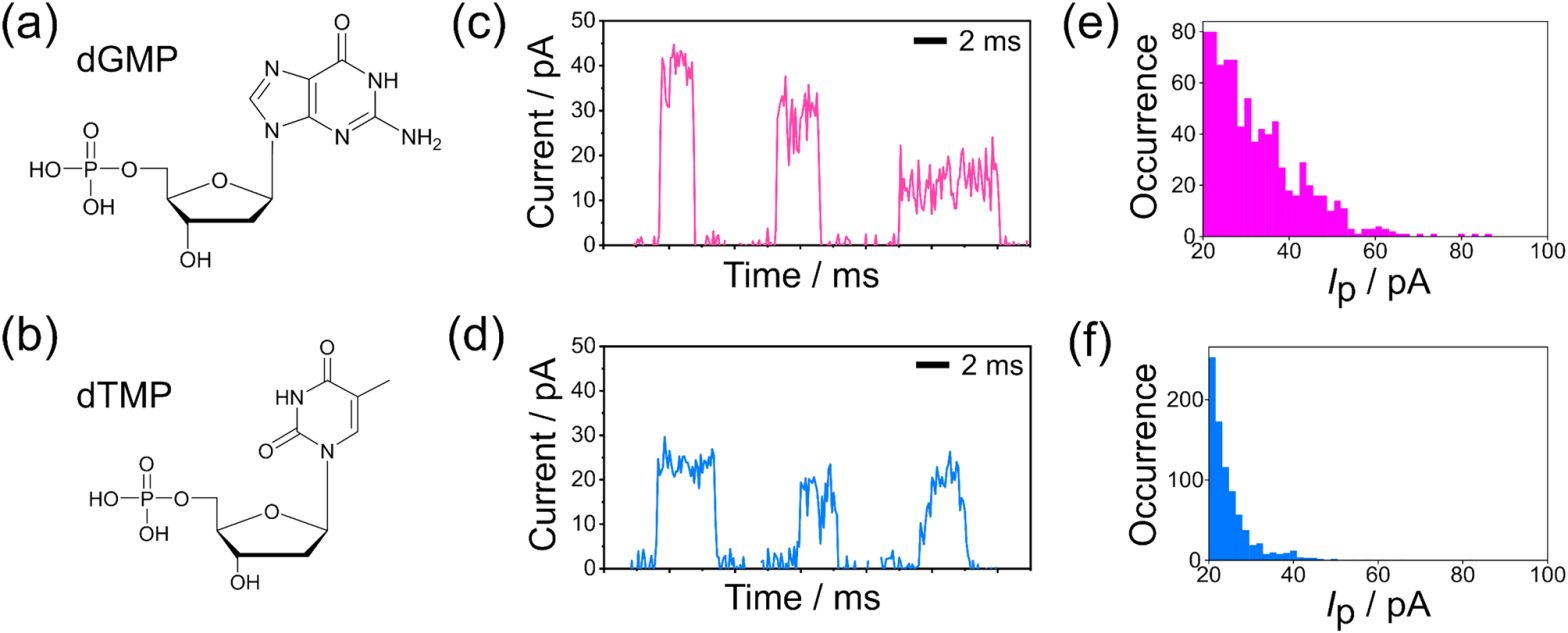
Results of dGMP and dTMP single-molecule measurements. (**a**), (**b**) Molecular structure of dGMP and dTMP, respectively. (**c**), (**d**) Three individual current pulses of dGMP and dTMP. (**e**), (**f**) Histograms of the maximum current (*I*_p_) for dGMP and dTMP, respectively. Each current is measured under a bias of 100 mV.

The target molecules in this study are two DNA nucleotides, deoxyguanosine monophosphate (dGMP) and thymidine monophosphate (dTMP). These targets were selected as model systems for single-molecule signal identification using machine learning rather than for their applicability in identifying mixtures of two molecules. Nucleotides can be identified by single-molecule measurements and have been previously reported as target molecules in various studies^15,17,36^. Figure 2a,b show the molecular structures of dGMP and dTMP, respectively. As shown in Fig. 2c,d, a current pulse signal is generated when an individual molecule passes through the nanogap. Figure 2c,d show histograms of the maximum current (*I*_p_) values. The average currents for dGMP and dTMP are 32 pA and 25 pA under a 100 mV bias voltage for dGMP and dTMP, respectively. dGMP exhibits a higher conductance than dTMP does because its HOMO level is closer to the Au Fermi level^39^, which is the conduction orbital for dGMP rather than for dTMP. Although the average conductance of the two molecules shows a clear difference, their *I*_p_ histograms exhibit an overlap. Both histograms exhibit low-current signals at 20 pA. The low-current signal was caused the single-molecule bridging structure between the nanogap. Electron transport via lower molecular orbital of ribose sugar cause lower current^40^. The large overlapping indicates that relying solely on histogram-based analysis methods that depend on *I*_p_ is insufficient for accurate discrimination and that the use of machine learning is necessary.

**Figure 3:**
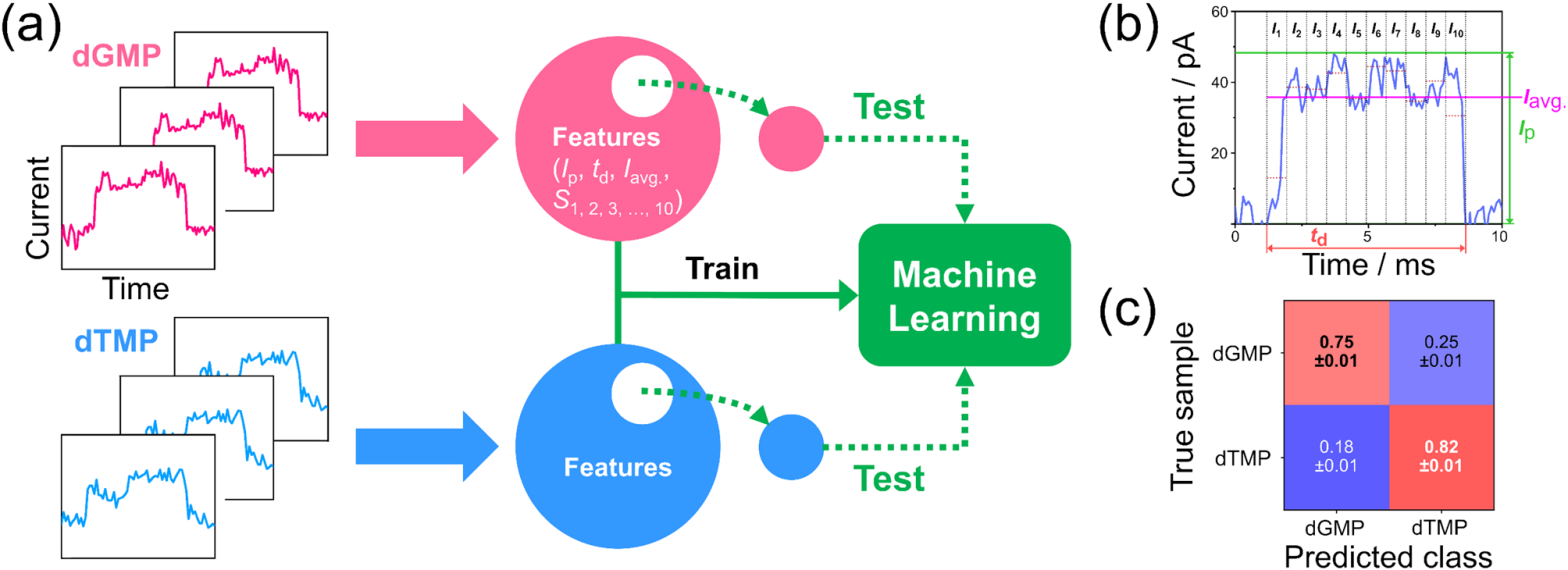
Conventional machine-learning training methods and identification results. (**a**) Training process of machine learning for pure solutions using the conventional method. Features includes factors such as peak current (*I*_p_), duration (*t*_d_), average current (*I*_avg._), and 10-dimensional normalized current for each pulse signal. (**b**) Single-molecule individual current pulse (blue solid line) and definitions of the features. The black dashed lines show the area of the current pulse divided into ten parts along the time axis. The average current values (red dashed lines) of each portion divided from *I*_1_ to *I*_10_ are 13.2, 38.3, 38.0, 43.5, 35.4, 44.1, 42.0, 34.3, 39.0, and 30.8 pA, respectively. *S*_*i*_ means *I*_*i*_ normalized with respect to *I*_p_. The green, red, and pink solid lines represent *I*_p_, *t*_d_, and *I*_avg._, respectively. (**c**) Confusion matrix of dGMP and dTMP predictions in pure solutions.

**Figure 4:**
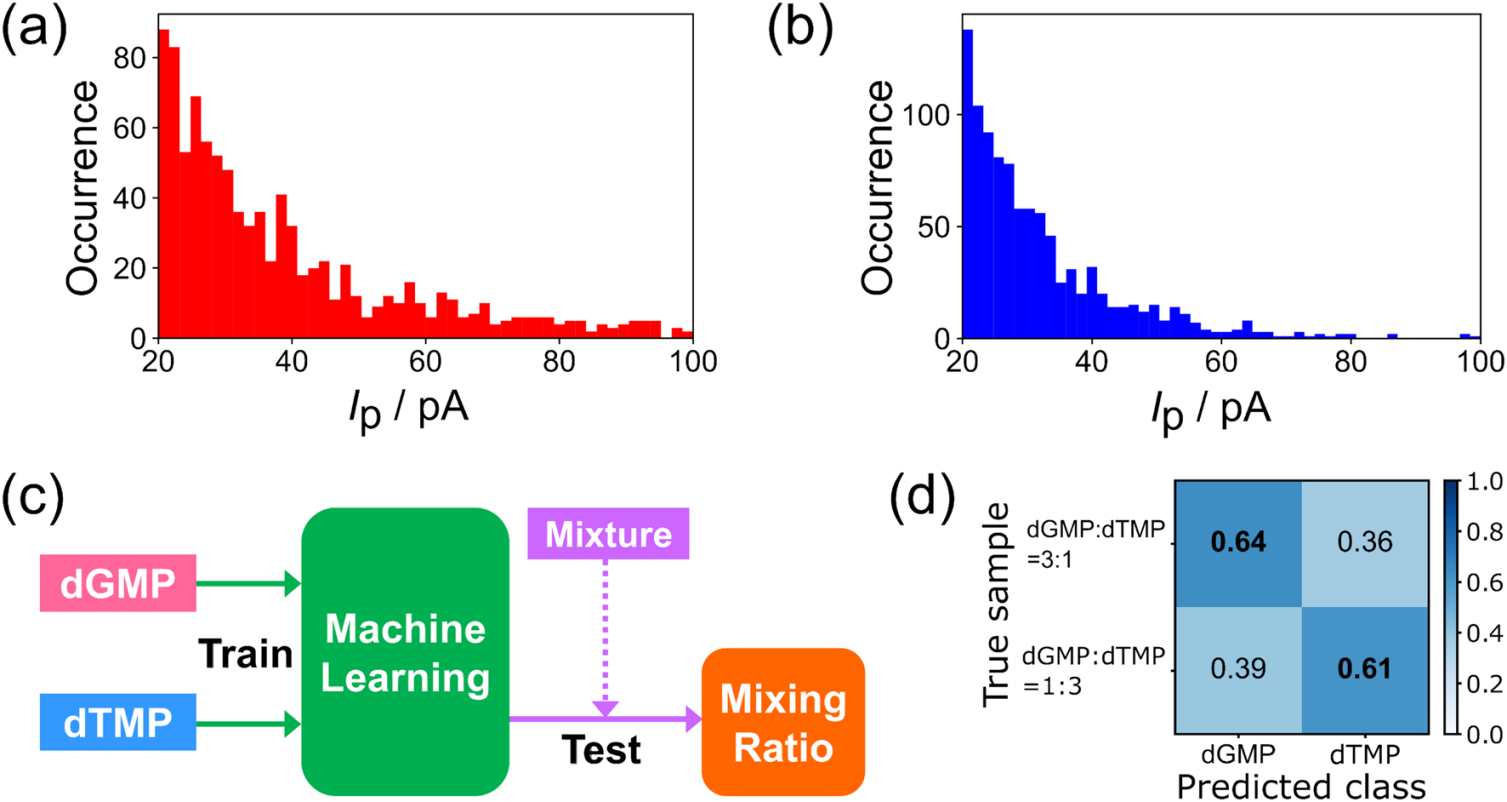
Process and results of predicting the mixing ratio of the target using the classifier trained on the current signal of the molecule in the previous step. (**a**), (**b**) *I*_p_ histograms measured in two mixtures, dGMP:dTMP = 3:1 and dGMP:dTMP = 1:3, respectively. (**c**) The process of identifying the current signal of mixtures using the machine-learning classifier trained on the current signal of each target to predict the mixing ratio. (**d**) The results of predicting the mixing ratio of mixtures based on trained data.

As a comparison to the proposed method, the mixing ratio of the mixture was predicted using a conventional machine-learning-based classification method. In the conventional method, the machine-learning classifier is first trained from the single-molecule current signals obtained from measurements of each single-target solution with the label of molecular names. The machine-learning classifier then identifies the current signals obtained from the mixture based on the learned characteristics of each molecular signal. Finally, each predicted molecular label of the mixed solution data is counted, and the concentration ratio is determined as the ratio of the number of signals for each molecule. Fig. 3a shows the validation process of the machine-learning classifier training. The machine-learning validation process consists of mechanically controllable break junction (MCBJ) measurement, signal extraction, feature extraction, training, and identification. In this study, 13-dimensional vectors consisting of *I*_p_, duration time (*t*_d_), and the 10-dimensional normalized current factor, which were used in previously reported methods, are used as features^20,26,35,36^. The 10-dimensional normalized current factors are defined as the average current value normalized by the maximum current value of each of the 10-time sections, as shown in Fig. 3b. A 10-fold cross-validation (CV) method was used for verification, training, and prediction as shown in Fig. S1 in Supplementary information. In 10-fold CV, all data are divided into ten subsets, and one subset is used as the testing data, whereas the identification is trained by the other subsets in a 10-time loop to ensure that all data are tested once. The validation results for the two molecules measured in pure solutions are presented in the confusion matrix shown in Fig. 3c. The F-measure, a performance index of classification, is 0.78. This approach demonstrates the identifiability of a machine-learning classifier trained on data measured from solutions containing only a single chemical species. To confirm the discriminative ability of the classifier, the mixing ratio of the target was predicted using a machine-learning classifier that learned the current signal of each molecule in the previous step. Figure 4a,b show the histograms of *I*_p_ measured in the two mixtures dGMP:dTMP = 3:1 and dGMP:dTMP = 1:3, respectively. The dGMP:dTMP = 3:1 solution, which contains more of the more-conductive dGMP, shows higher conductance than the dGMP:dTMP = 1:3 solution, which contains more of the less-conductive dTMP. Figure 4c shows the process of identifying the current signals obtained in the mixture using the machine-learning classifier trained from the current signals of each target in the previous step to predict the mixture ratio. Using this process, the machine-learning classifier predicted mixing ratios of 1.7:1 and 1:1.6 for the signals obtained from the dGMP:dTMP = 3:1 and dGMP:dTMP = 1:3 solutions, respectively, as shown in Fig. 4d. As shown in Fig. 3c, the identification accuracy of each nucleotide varies individually, which can result in an underestimation of the prediction ratio of abundant nucleotides.

**Figure 5:**
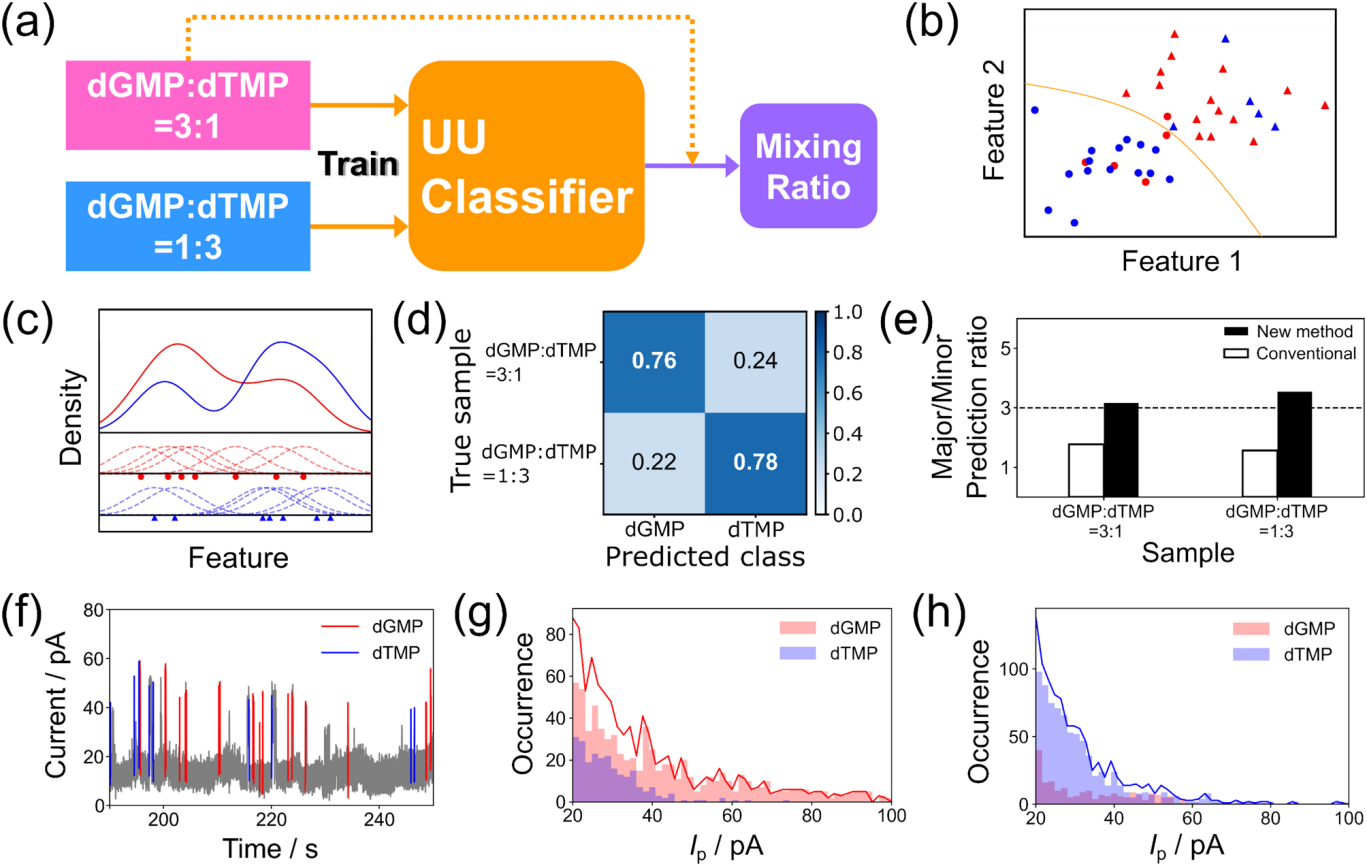
(**a**) Process of training and identifying with data from mixtures only. (**b**) Schematic image of UUC. The red and blue colors represent two types of mixtures with different concentrations of the two classes. The circles and triangles represent each class. The UUC method determines the orange curve, which represents the boundary between two classes. (**c**) Schematic image of the KDE for estimating the probability density function in the feature space. The red and blue dots and dashed lines indicate the data points and their Gaussian kernel, respectively. The solid curves represent the sum of the dashed lines, which represents the kernel density estimate. (**d**) The result of predicting the mixing ratio of two mixtures with data trained on the mixture only. (**e**) Comparison of the performance of the new and old methods with respect to the prediction ratio. (**f**) The current profile resulting from identifying the signal of each single molecule individually (in dGMP:dTMP = 3:1 solution). (**g**), (**h**) *I*_p_ histograms based on the identification results of the dGMP:dTMP = 3:1 and dGMP:dTMP = 1:3 solutions, respectively. The red and blue bars represent the histograms predicted as dGMP and dTMP, respectively. The solid lines represent the sum of the two histograms.

The main goal of this research is to develop a method to distinguish between the two molecules from the data measured using only mixed solutions. The relationship between the concentrations of the two mixtures, that is, solutions containing more dGMP or dTMP, is known. The measurement and identification processes for this new concept are illustrated in Fig. 5a. The discriminative boundaries of the two molecules were estimated directly from the data obtained from the two mixtures with unlabeled data and unlabeled data classification (UUC) based on kernel density estimation (KDE)^41^. Fig. 5b shows a conceptual diagram of UUC, a method for determining discriminant boundaries from data in which the two classes are mixed in different concentrations. In Fig. 5b, the blue and red colors represent two types of mixtures. Both solutions contain different concentrations of the two classes. The classes are unknown in advance. The purpose of UUC is to distinguish between these two classes based on which class is more abundant in the solution. KDE is a non-parametric statistical technique used to estimate the probability density function in a feature space directly from observed data, as shown in Fig. 5c. Intuitively, KDE calculates the probability density by adding the Gaussian kernels obtained from each observed data point, similar to the manner in which a histogram is created by adding data points. This method can obtain a smooth probability density distribution with fewer data than that of a histogram. In this study, the Gaussian kernel was centered on the observed data points. In the UUC method used in this study, the probability density distributions of the two classes were determined by KDE through correction. This method is proposed for a situation in which one of the data points contains only positive classes. However, because the proposed method is based on the principle that regions of higher concentration exhibit higher probability densities, it can also be applied to two unlabeled data mixtures with known concentration relationships. For comparison with the conventional method, identification was performed with the same features extracted from the same dataset as that described in the previous section. The UUC machine learning classifier was trained using only the signals from the mixtures and predicted the molecules, and the results are presented in Fig. 5d. The ratios of signals corresponding to 3:1 and 1:3 ratios of dGMP:dTMP were predicted to be 3.2:1 and 1:3.5, respectively. The performance of the new identification method proposed in this study is compared with that of conventional methods, as shown in Fig. 5e. The electronic structures of the electrodes affect single-molecule conductance. Electronic structure variation due to molecular adsorption on the electrode surface or different geometries of the electrodes may affect single-molecule signals^42–45^. A wide variety of machine learning methods have been developed in recent years. Unsupervised learning is applicable to the identification of data without explicit labels, as is supervised learning. This method has been applied to the discrimination of *I–z* traces of single-molecule measurements^34^. However, conventional unsupervised learning methods cannot adequately identify the experimental data from the two solutions, as shown in SI.5. The new UUC method can discriminate between two molecules by measuring only the mixtures. The method is assumed to prevent the propagation of errors owing to environmental changes and cause higher accuracy discrimination than conventional methods. Figure 5f shows the current profile of the dGMP:dTMP = 3:1 solution with the molecular prediction results obtained by the UUC method. The red and blue signals denote dGMP- and dTMP-derived signals, respectively. The signals obtained from the mixtures can be discriminated individually.

In the previous section, the conductance histograms of individual nucleotides (Fig. 2) showed that dGMP has a higher conductance. Focusing on the individual signals identified, the dGMP signal does not always show a higher conductance than the dTMP signal. Machine-learning algorithms can differentiate between signals based on both the conductance and signal shape. This is because the current histograms of the identified results are statistically analyzed. The *I*_p_ histograms of the identified results of the signals obtained from dGMP:dTMP = 3:1 and dGMP:dTMP = 1:3 solutions are shown in Fig. 5g,h, respectively. The red and blue bars represent histograms predicted as dGMP and dTMP, respectively. The histograms confirm that the UUC method can predict mixing ratios and that dGMP has a higher conductance than dTMP. This agrees with the results of the pure-solution measurement. Notably, this new method enables the determination of concentration ratios using only two mixture solutions of unknown concentrations. This technique is assumed to be applicable to molecular detection methods. For example, this technique can be applied to determine the concentration ratio of a molecule in a biological sample containing a foreign material by comparing it to a normal sample and a positive/negative sample with a control that promotes or inhibits the molecule of interest or by measuring the concentration of the molecule of interest in a sample of unknown concentration and a sample to which a reference sample is added. The concentration ratio of the molecule of interest can also be determined from positive/negative samples of the molecule of interest with a control that promotes or inhibits the molecule of interest.

## Conclusions

In this study, we developed a new method to identify molecules using single-molecule measurement of only mixed solutions and a discrimination method for two types of unlabeled data using kernel density estimation. Compared to the traditional method, our approach showed improved accuracy in predicting the composition of mixed solutions. The technique developed in this study for identifying target molecules in mixed solutions without individual sample training is expected to have broad applications for various molecules in the field of single-molecule measurement.

## Methods

### Preparation of sample solutions

Deoxyguanosine monophosphate (dGMP, Sigma-Aldrich) and deoxythymidine monophosphate (dGTP, Sigma-Aldrich) were diluted in Milli-Q water without any further purification process. The concentration of each solution of dGMP and dTMP used in the measurement was 10 μM. Measurements of dGMP:dTMP = 3:1 used the mixture of 750 μM dGMP and 250 μM dTMP, and measurements of dGMP:dTMP = 1:3 used the solution of 250 μM dGMP and 750 μM dTMP. Polydimethylsiloxane (PDMS) wells were fabricated and treated with an oxygen plasma for 10 s, attached to the MCBJ nanogap electrode device, and treated in the vacuum oven at 90 °C for 60 min.

### Device fabrication

The MCBJ technique was applied to form gold nanogaps. The gold wires were deposited on the flexible silicon substrate. First, polyimide thin-film was formed as an insulating layer on the silicon substrate. Tens of nanometer-wide patterns were fabricated using electron beam lithography, and the gold wires were deposited on the patterns using plasma-enhanced chemical vapor deposition. Finally, the polyimide layer was dry etched to form the gold wire bridge. The gold wire substrate was installed in the MCBJ system and the current change was monitored until the wires were mechanically broken due to repeated bending by three-point bending and a sharp current drop appeared. During this process, the current was measured using the piezoelectric device to precisely control the gap width in real time and fine-tune the piezo-adjusted pushing rod.

### Electrical measurement of single-molecule

The solutions were injected into PDMS well attached to the MCBJ device. A voltage of 100 mV was applied to the solution electrode for 5 min. Before every individual measurement, a control experiment was performed by injecting only Milli-Q water. The interelectrode distance d of the nanogap was fixed at 0.58, 0.56, and 0.54 nm by the MCBJ technique.

### Machine learning analysis

Each of the 830 pulse signals was trained and classified with supervised machine learning of the Random Forest (RF) classifier in scikit-learn version 0.24.2^46^. In validation process, the 10-fold CV was performed and its average and standard deviation values provided the classification ratios and errors. The errors are standard deviation of 10-time classification. In mixed solution analysis, the RF supervised machine learning classifier was trained with 1000 dGMP and dTMP signals each. Signals with *I*_p_ > 20 pA and *t*_d_ > 1 ms were analyzed. The signals from the mixtures were classified one by one with the trained classifier. The analysis was performed using Python 3.10.4. UUC and weighted KDE source codes were prepared by ourselves using Python 3.10.4. The 1000 signals and features from mixtures are same to conventional methods. Gaussian kernel was adopted. The bandwidth is determined by Silverman’s rule^41^.

## Supplementary Information

Supplementary Information.

## Data Availability

The data that support the findings of this study are available from the corresponding author upon reasonable request. Correspondence and requests for materials should be addressed to M.T.
