# Supplementary material for: Direct biomolecule discrimination in mixed samples using nanogap-based single-molecule electrical measurement
Source: Sci Rep. 2023 Jun 5;13:9103. doi: 10.1038/s41598-023-35724-1 (PMC10241884; doi:10.1038/s41598-023-35724-1)
Supplement: Supplementary file 1 — Supplementary Information. [file 41598_2023_35724_MOESM1_ESM.docx]

*Supplementary Information for*

**Direct Biomolecule Discrimination in Mixed Samples using Nanogap-Based Single-Molecule Electrical Measurement**

Jiho Ryu^1^, Yuki Komoto^1,2,3^, Takahito Ohshiro^1^, Masateru Taniguchi^1^*

^1^SANKEN, Osaka University, 8-1 Mihogaoka, Ibaraki, Osaka, 567-0047, Japan

^2^Artificial Intelligence Research Center, Osaka University, Ibaraki, Osaka 567-0047, Japan

^3^Integrated Frontier Research for Medical Science Division, Institute for Open and Transdisciplinary Research Initiative (OTRI), Osaka University, Ibaraki, Osaka 567-0047, Japan

Correspondence to: taniguti@sanken.osaka-u.ac.jp

**Table of Contents**

1. Estimation of interelectrode distance………………………………………..2

2. Details of 10-fold Cross-Validation…...…….………………..……………..3

3. Current profiles in each solution…..……….………………..…..…………..4

4. Histograms of the dwell time of the current pulse....…………………………5

5. Unsupervised learning and comparison of clustering algorithms..…………6

**1. Estimation of interelectrode distance**

The distance between the nano-gap electrode and electrode was estimated using the current equation for direct tunneling current:

$I=const exp\left( -\frac{4\pi}{h}\sqrt{2mw}l \right)$.

In this equation, *h*, *m*, *w*, and *l* represent Planck’s constant, electron mass, work function of the gold electrode, and gap distance, respectively. Here, we used electron mass of 9.1 ×10^-31^ kg as *m*, and the work function of Au (111) as *w*. For accurate estimation, the effective mass and work function of the (111) surface rather than the gold nanogap were used. Furthermore, the widening of the inelastic gold gap immediately after breaking the atomic bond was not considered. Hence, the nano-gaps applied in the experiment are larger than the target values of 0.58, 0.56, and 0.54 nm.

**2. Details of 10-fold cross-validation**


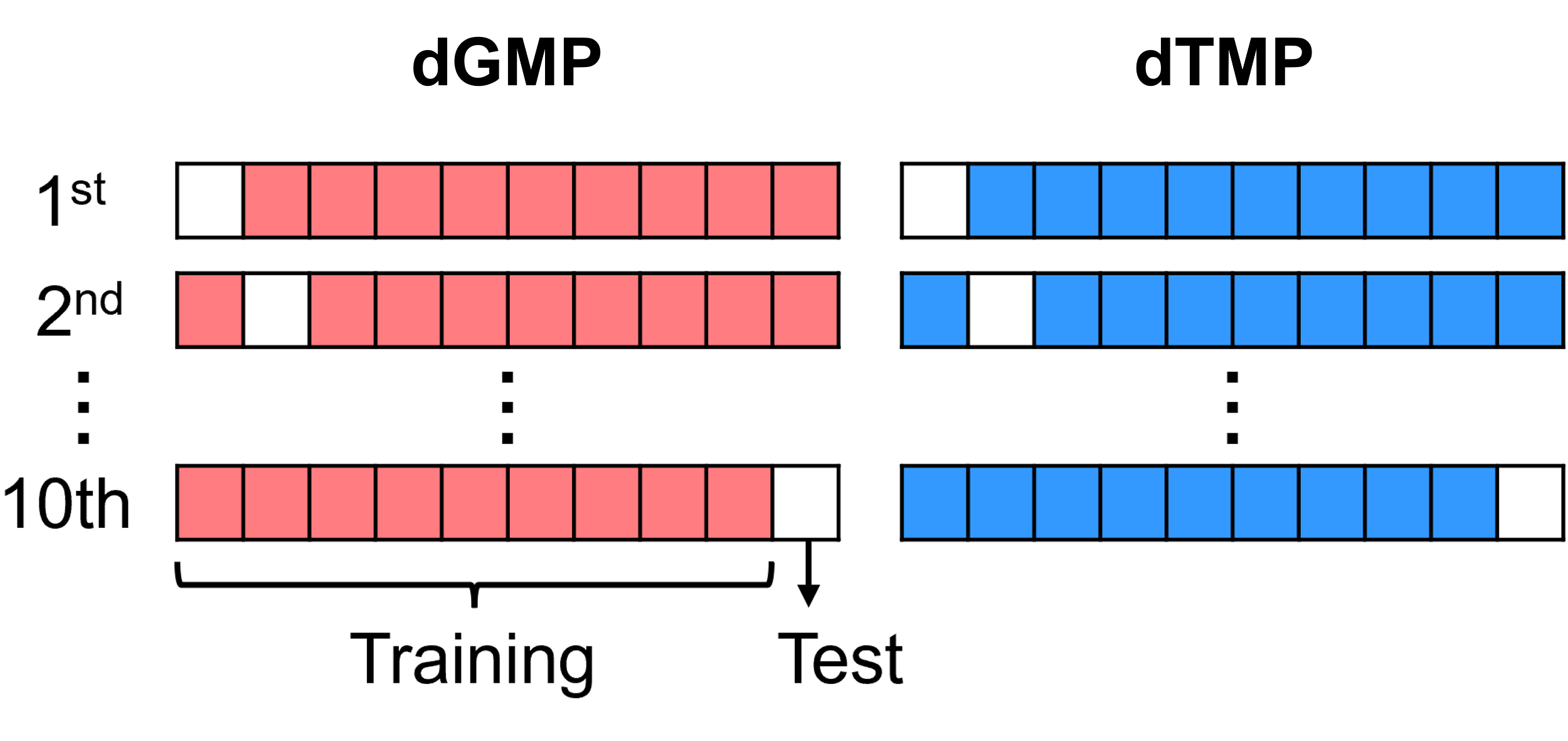


**Figure S1**. Schematic image of 10-fold cross-validation (CV). 10-fold CV randomly divides the dataset into 10 equal-sized subsets. In each fold, one subset is held out as the test set, while the remaining 9 subsets are used for training. This process is repeated 10 times, with each subset used once for validation. Model performance is estimated by averaging the results of 10 validation processes. This technique ensures that all observations are used for both training and validation, with each observation used for validation exactly once.

**3. The current profiles in each solution**

**
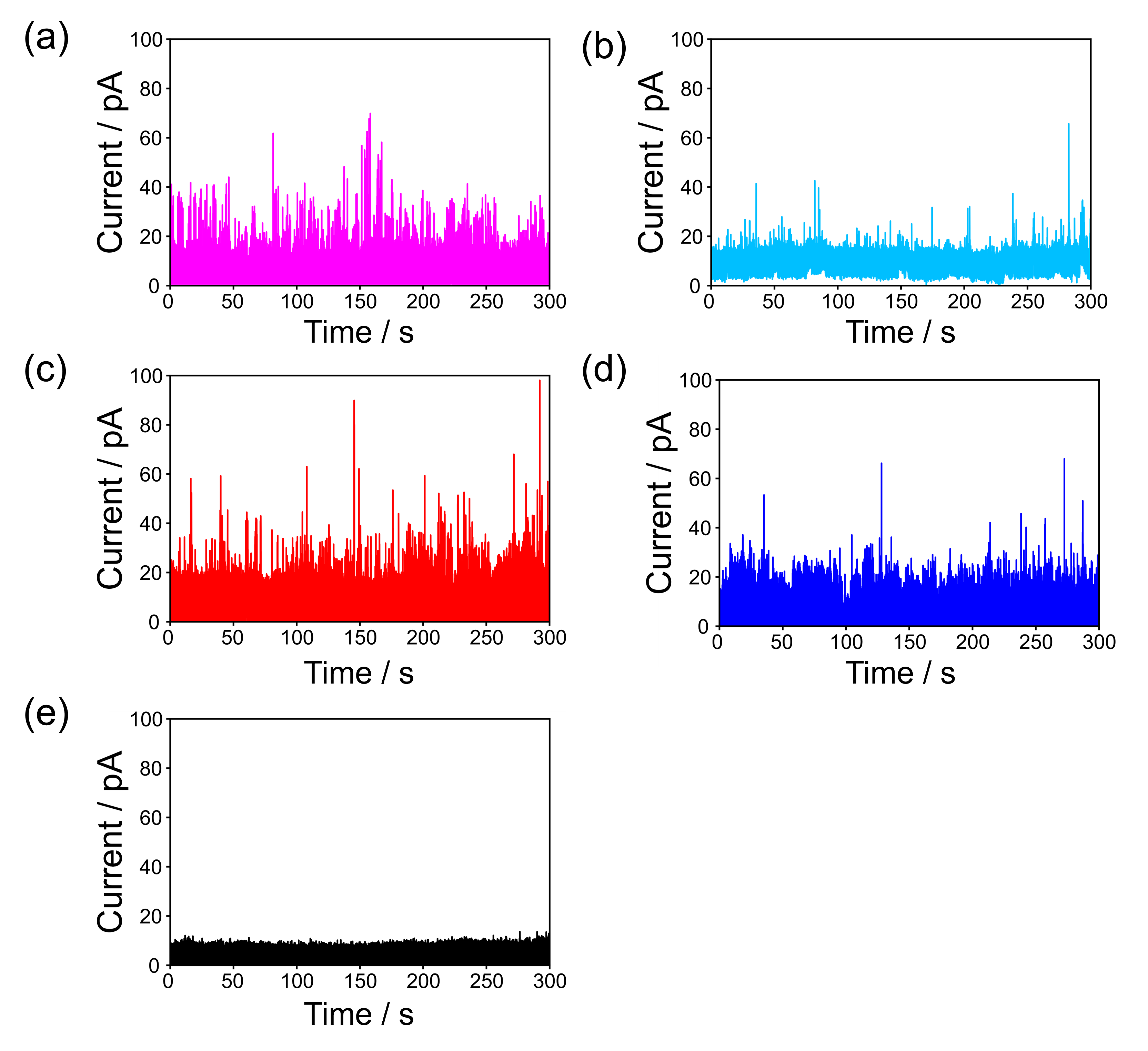
**

**Figure S2**. The current profiles of each measurement. (a) dGMP solution, (b) dTMP solution, (c) dGMP:dTMP= 3:1 solution, (d) dGMP:dTMP= 1:3 solution, (e) Milli-Q water as the blank solution. All solutions used in the measurements were prepared using deionized Milli-Q water.

**4. Histogram of the dwell time of the current pulse**


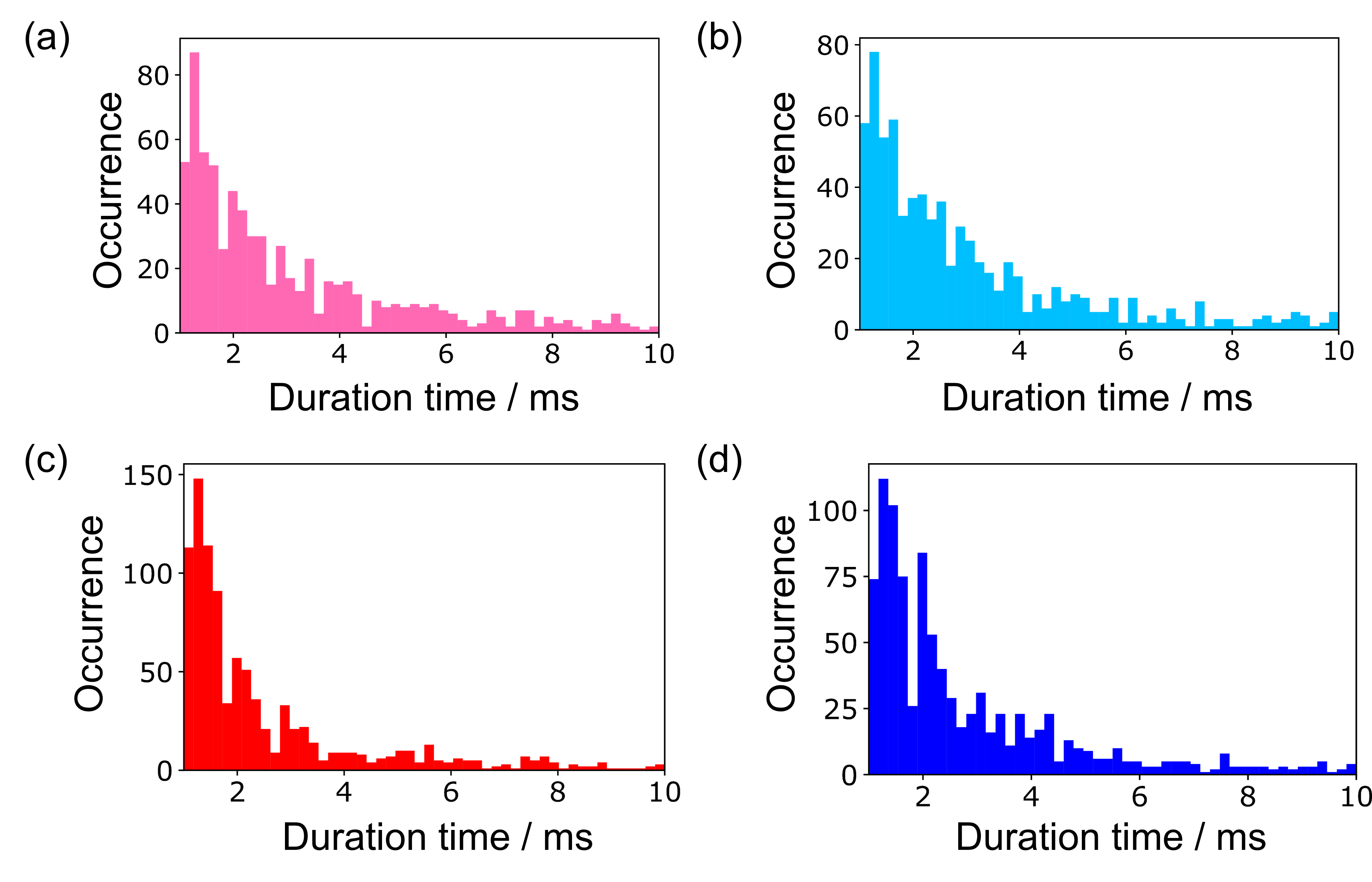


**Figure S3**. Histograms of the duration of the current pulse for each measurement. (a) dGMP solution, (b) dTMP solution, (c) dGMP:dTMP= 3:1 solution, (d) dGMP:dTMP= 1:3 solution.

**5. Unsupervised learning and comparison of clustering algorithms**


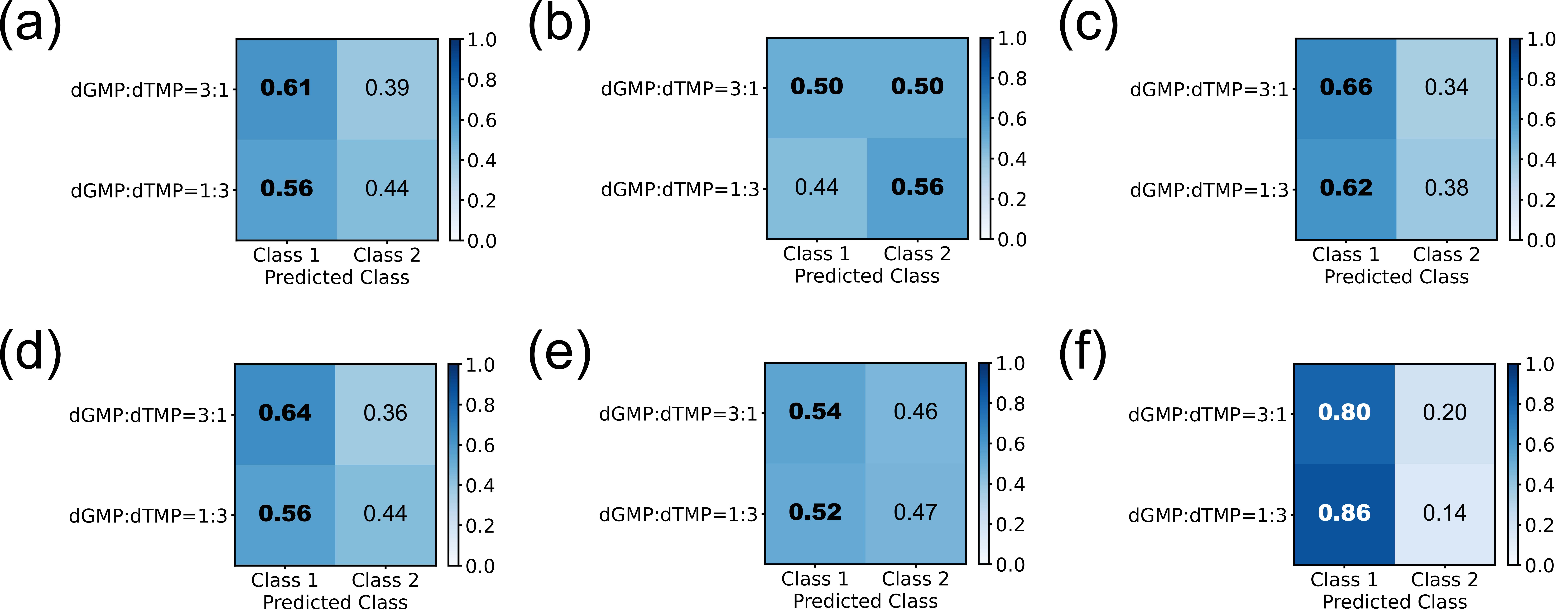


**Figure S4.** Discrimination results with unsupervised machine learning. (a-c) Discrimination results without PCA and (d-f) with PCA. The clustering algorithms are (a d) *k*-means clustering, (b, e) spectral clustering, and (c, f) GMM clustering.

For comparison, the molecular ratios of the mixtures were determined using existing unsupervised learning. Three clustering algorithms, k-means, spectral clustering, Gaussian mixture model (GMM) were applied from sci-kit learn version '0.24.2'.^49^ These algorithms require the number of clusters in advance.

The number of clusters: n_clusters is set to 2, “eigen_solver=’arpack’, affinity=’nearest_neighbors’" were chosen for spectral clustering parameters. Other parameters were default. The three clustering methods were also performed with and without dimensionality reduction by principal component analysis (PCA) as a pre-processing step. The dimension was reduced to two by PCA.

Fig. S4 represents the discrimination results. No method cannot discriminate adequately. The shape of clusters is defined in advance for the k-means and GMM algorithms. In spectral clustering, the clustering is performed with similarity between the data. These methods may be not suitable for two classes clustering of complex distributional data. Our method is kernel density estimation-based and designed for learning with unlabeled data. Our method achieves better performance for complex features.
